# Risk of Stroke Hospitalization After Infertility Treatment

**DOI:** 10.1001/jamanetworkopen.2023.31470

**Published:** 2023-08-30

**Authors:** Devika Sachdev, Rei Yamada, Rachel Lee, Mark V. Sauer, Cande V. Ananth

**Affiliations:** 1 Department of Obstetrics, Gynecology, and Reproductive Sciences, Rutgers Robert Wood Johnson Medical School, New Brunswick, New Jersey; 2 Division of Epidemiology and Biostatistics, Department of Obstetrics, Gynecology, and Reproductive Sciences, Rutgers Robert Wood Johnson Medical School, New Brunswick, New Jersey; 3 Cardiovascular Institute of New Jersey, Rutgers Robert Wood Johnson Medical School, New Brunswick, New Jersey; 4 Department of Biostatistics and Epidemiology, Rutgers School of Public Health, Piscataway, New Jersey; 5 Environmental and Occupational Health Sciences Institute, Rutgers Robert Wood Johnson Medical School, Piscataway, New Jersey

## Abstract

**Importance:**

Stroke accounts for 7% of pregnancy-related deaths in the US. As the use of infertility treatment is increasing, many studies have sought to characterize the association of infertility treatment with the risk of stroke with mixed results.

**Objective:**

To evaluate the risk of hospitalization from hemorrhagic and ischemic strokes in patients who underwent infertility treatment.

**Design, Setting, and Participants:**

This population-based, retrospective cohort study used data abstracted from the Nationwide Readmissions Database, which stores data from all-payer hospital inpatient stays from 28 states across the US, from 2010 and 2018. Eligible participants included individuals aged 15 to 54 who had a hospital delivery from January to November in a given calendar year, and any subsequent hospitalizations from January to December in the same calendar year of delivery during the study period. Statistical analysis was performed between November 2022 and April 2023.

**Exposure:**

Hospital delivery after infertility treatment (ie, intrauterine insemination, assisted reproductive technology, fertility preservation procedures, or use of a gestational carrier) or after spontaneous conception.

**Main Outcomes and Measures:**

The primary outcome was hospitalization for nonfatal stroke (either ischemic or hemorrhagic stroke) within the first calendar year after delivery. Secondary outcomes included risk of stroke hospitalization at less than 30 days, less than 60 days, less than 90 days, and less than 180 days post partum. Cox proportional hazards regression models were used to estimate associations, which were expressed as hazard ratios (HRs), adjusted for confounders. Effect size estimates were corrected for biases due to exposure misclassification, selection, and unmeasured confounding through a probabilistic bias analysis.

**Results:**

Of 31 339 991 patients, 287 813 (0.9%; median [IQR] age, 32.1 [28.5-35.8] years) underwent infertility treatment and 31 052 178 (99.1%; median [IQR] age, 27.7 [23.1-32.0] years) delivered after spontaneous conception. The rate of stroke hospitalization within 12 months of delivery was 37 hospitalizations per 100 000 people (105 patients) among those who received infertility treatment and 29 hospitalizations per 100 000 people (9027 patients) among those who delivered after spontaneous conception (rate difference, 8 hospitalizations per 100 000 people; 95% CI, −6 to 21 hospitalizations per 100 000 people; HR, 1.66; 95% CI, 1.17 to 2.35). The risk of hospitalization for hemorrhagic stroke (adjusted HR, 2.02; 95% CI, 1.13 to 3.61) was greater than that for ischemic stroke (adjusted HR, 1.55; 95% CI, 1.01 to 2.39). The risk of stroke hospitalization increased as the time between delivery and hospitalization for stroke increased, particularly for hemorrhagic strokes. In general, these associations became larger for hemorrhagic stroke and smaller for ischemic stroke following correction for biases.

**Conclusions and Relevance:**

In this cohort study, infertility treatment was associated with an increased risk of stroke-related hospitalization within 12 months of delivery; this risk was evident as early as 30 days after delivery. Timely follow-up in the immediate days post partum and continued long-term follow-up should be considered to mitigate stroke risk.

## Key Points

**Question:**

Is receipt of infertility treatment associated with stroke hospitalization?

**Findings:**

In this cohort study of 31 339 991 pregnant individuals who delivered between 2010 and 2018, compared with those who did not receive infertility treatment, those who received infertility treatment had an increased risk of stroke hospitalization within 12 months of delivery, with the risk of hospitalization for hemorrhagic stroke being substantially greater than that for ischemic stroke.

**Meaning:**

These findings suggest that infertility treatment is associated with an increased risk of stroke hospitalization within 12 months of delivery; therefore, optimal screening for risk and timely follow-up should be considered to mitigate factors associated with stroke in the antepartum and postpartum periods.

## Teaser

This cohort study compares the risk of hospitalization for stroke among individuals who delivered after receiving infertility treatment vs individuals who delivered after spontaneous conception.

## Introduction

Infertility treatment has grown at a rapid pace over recent years given substantial advancements in technology, the development of new medications and protocols, and overall improved access to care. The use of assisted reproductive technology (ART) has approximately doubled in the past decade, and roughly 2% of all live births in the US involve some method of ART.^1^ Although treatments involved are generally safe and well tolerated with a low propensity for severe complications, a growing body of evidence points to certain adverse pregnancy and medical outcomes associated with infertility treatments.^2,3,4^ Consequently, closer attention has been paid recently to these possible adverse outcomes, including heart disease and stroke.

Cardiovascular disease (CVD) remains the leading cause of death in women, with 1 in 3 deaths attributable to CVD each year.^5^ Stroke is the third leading cause of death among both men and women.^6,7^ Studies^5,8^ indicate 1 in 5 women is at risk of developing a stroke in their lifetime, and evidence suggests that many do not know the health factors that put them at risk for stroke or other CVD. However, the stroke mortality rate in the US has declined substantially over the last 4 decades.^9^ This decline has been steeper for ischemic stroke rather than hemorrhagic stroke subtypes.

Even more alarming is that CVD is now the leading cause of maternal mortality in the US with over 26.5% of pregnancy-related deaths attributable to this condition.^10,11,12,13^ Specifically, stroke accounts for approximately 7% of pregnancy-related deaths in the US.^13^ Certain health factors may predispose a pregnant patient to the development of stroke, such as preterm delivery, fetal growth restriction, gestational diabetes, and hypertensive complications.^7,10^

What is less known, however, is whether infertility treatment is associated with pregnancy-related stroke incidence. In a retrospective cohort study^14^ of 4710 women from Taiwan between 2000 and 2010, infertility medications were associated with an increased risk of deep venous thrombosis; however, the authors found a lower risk of ischemic stroke. Additionally, a population-based Canadian cohort study^15^ (1993 to 2010) concluded that infertility treatment was not associated with an increased risk of CVD. Other studies^16,17^ have seen increased trends in the development of stroke after infertility treatment, but a 2017 meta-analysis^17^ stated that the small number of studies available prohibits any substantial conclusions.

The association of infertility treatment with nonfatal stroke risk is even less apparent. A proposed theory is that the increased risk of hypertension and gestational diabetes (2 well-known cardiovascular factors associated with risk of stroke) in those undergoing infertility treatment may increase future stroke risk.^15,18^ Other theories as to why there may be an association of infertility treatment with stroke risk include direct endothelial cell damage after infertility treatment and release of prothrombotic factors after ovarian hyperstimulation because supraphysiologic doses of estrogen are often required in vitro fertilization (IVF) protocols.^15,19,20^

Given these potential reasons why infertility treatment may be associated with stroke risk, we hypothesized that treatment for infertility is associated with an increased risk of both hemorrhagic and ischemic stroke. We tested this hypothesis in a large cohort of pregnant individuals with hospital deliveries in the US who were subsequently hospitalized up to 1 year post partum from a stroke complication. We used a cutoff of 1 year given that the greatest risk of pregnancy-related stroke is in the peripartum period^21^ but the risk is still apparent up to 12 weeks post partum^22^ and likely further beyond.

## Methods

This retrospective cohort study followed the Strengthening the Reporting of Observational Studies in Epidemiology (STROBE) reporting guideline^23^ and did not require institutional review board approval or informed consent because the data used in the study were deidentified in accordance with 45 CFR § 46. The Nationwide Readmissions Database (NRD) was used to obtain our data for this study from 2010 to 2018. This database was developed by the Agency for Healthcare Research and Quality for the Healthcare Cost and Utilization Project (HCUP)^24^ and contains all-payer hospital inpatient stays that can be used to generate national estimates of readmissions.^25^ The NRD is drawn from the HCUP state inpatient databases containing verified patient linkage numbers that can be used to identify a patient across hospitals within a state.^25^ In 2018, 28 HCUP partner states contributed to the NRD.^25^ To reflect the target universe, weights were calculated and applied through poststratification by hospital and discharge characteristics. Application of these weights in statistical analyses ensures proper variance estimation of parameters and affords generalizability of findings related to hospital discharges. Outcomes reported include national readmission rates, reasons for returning to the hospital for care, and hospital costs for discharges with and without readmission.

The cohort was obtained by using *International Classification of Diseases, Ninth Revision, Clinical Modification* (*ICD-9-CM*) and *International Statistical Classification of Diseases, Tenth Revision, Clinical Modification* (*ICD-10-CM*) diagnosis codes and procedure coding system (PCS) to identify patients who had delivered in a hospital, with or without infertility treatment. Between 2010 and the first 3 quarters of 2015, the NRD included diagnosis and procedure codes using the *ICD-9-CM* and PCS coding system. From the fourth quarter of 2015 until 2018, the NRD included diagnosis and procedure codes using the *ICD-10-CM* and PCS coding system. The *ICD-9-CM* and *ICD-10-CM* codes used in the study are shown in the eTable in Supplement 1.

### Inclusion Criteria

The study included individuals aged 15 to 54 years who had a hospital delivery from January to November in a given calendar year, and any subsequent hospitalizations from January to December in the same calendar year of delivery. Deliveries were restricted from January through November to allow estimation of 30-day hospital admission for stroke. All deliveries were included regardless of plurality (ie, singleton and multiple births), delivery outcome (ie, live birth and stillbirths), or gestational age.

### Exclusion Criteria

Patients with any hospitalizations with CVD prior to delivery and/or during delivery and their subsequent hospitalizations were excluded from the cohort. Ectopic pregnancy, molar pregnancy, and abortive outcomes were also excluded.

### Exposure

The exposure group were patients who conceived after infertility treatment. Infertility treatment was broadly defined and included intrauterine insemination and ART, including IVF or gamete intrafallopian transfer, fertility preservation procedures, or use of a gestational carrier. For a full list of infertility treatment diagnosis codes, refer to the eTable in Supplement 1.

### Outcomes

The primary outcome was hospitalization for development of nonfatal stroke, defined as either ischemic or hemorrhagic, within 12 months post partum. Secondary outcomes included the risk of nonfatal stroke hospitalizations within 30, 60, 90, and 180 days post partum, and overall stroke mortality in those who underwent infertility treatment.

### Statistical Analysis

Infertility treatment status was determined by using all hospitalizations before, during, and after delivery. Given the complex sampling strategy of the NRD, all analyses were designed to account for sampling weights and hospital clusters (provided in the NRD). Associations of infertility treatment with hospitalizations for stroke were estimated by fitting Cox proportional hazards regression models with the interval between delivery and first stroke hospitalization as the person-time. We estimated the associations for any stroke hospitalization, as well as for ischemic and hemorrhagic stroke hospitalizations, within 1 year of delivery. We additionally fit discrete-time survival models to estimate stroke hospitalizations in relation to infertility treatment at less than 30 days, less than 60 days, less than 90 days, and less than 180 days since delivery. We estimated hazard ratios (HRs) and 95% CIs as the effect size measure before and after adjustment for confounders.

HRs were adjusted for the following confounders: maternal age, age-squared (both centered), multiple births, hospital bed size, hospital type, hospital teaching status, income quartile, insurance, and year of hospital discharge. We provide the distributions of comorbid conditions, including hypertensive complications of pregnancy (eg, chronic hypertension, preeclampsia with and without severe features, eclampsia, and superimposed preeclampsia), fetal growth restriction, placental abruption, gestational diabetes, stillbirth, multiple gestations, cesarean delivery, preterm delivery, and body mass index (calculated as weight in kilograms divided by height in meters squared) of 30 to 39.9 purely for descriptive purposes. These conditions were not adjusted in any of the associations since they were likely on the causal pathway, and an adjustment would likely result in a collider bias.^26,27^

We estimated person-time (in months) according to the discharge dates of delivery and stroke hospitalization. Although the discharge month and year were known, the date of discharge was not available in the NRD. Therefore, random integers were generated accounting for the number of days in each month and accounting for leap years. The event time was the discharge date at the first stroke admission after delivery. Patients who died or who were not hospitalized were censored.

All statistical analysis was undertaken using SAS statistical software version 9.4 (SAS Institute). The probabilistic bias analysis was accomplished using the episensr package in R statistical software version 4.3.0 (R Project for Statistical Computing).^28,29^ Statistical analysis was performed between November 2022 and April 2023.

#### Probabilistic Bias Analysis

Given the potential for exposure misclassification of infertility treatment,^30,31^ the potential for selection bias (with 28 of the 50 states contributing to the NRD), and the likelihood of the effect size estimates being affected by unmeasured confounding, we undertook a probabilistic bias analysis to simultaneously address all 3 biases.^32^ The sensitivity (0.03) and specificity (0.99) of infertility treatment in administrative databases were validated against the Society for Assisted Reproductive Technology Clinic Online Reporting System.^31^ To address exposure misclassification, we simulated data with the assumption that sensitivity of infertility treatment would vary between 0.4 and 0.8, and specificity of infertility treatment would vary between 0.99 and 1.0 under a uniform distribution and nondifferential exposure misclassification. The selection bias parameter was fixed at 0.56 (28 of 50 states). For unmeasured confounding, we assumed that the prevalence estimates of the confounder(s) among those with and without the infertility treatment would range between 0.05 and 0.25 (nondifferential) under a uniform distribution and the confounder-to-outcome rate ratio would range between 0.1 and 10.0 under a uniform distribution. These estimates to correct for unmeasured confounding bias were based on clinically plausible values. On the basis of these assumptions, we generated 500 000 simulation patterns and reported the median bias-corrected rate ratio with a 95% CI.

#### E-Values

The number of confounders is limited in the NRD data, thereby leaving the potential for associations to be biased from unmeasured confounding. An assessment of unmeasured confounding was based on the E-value.^33^ The E-value is a representation of how large an association of unmeasured confounders must be over that of the reported adjusted confounders to nullify the reported confounder-adjusted HR between the exposure and outcome, as well as move the 95% CI of the HR closest to the null to cross the null.^34^ In other words, a large E-value (relative to the confounder-adjusted effect size estimate) suggests that the likelihood of unmeasured confounding to have biased the reported HRs is likely small.

## Results

Of 31 339 991 patients who delivered between 2010 and 2018, 287 813 (0.9%; median [IQR] age, 32.1 [28.5-35.8] years) delivered after receiving infertility treatment and 31 052 178 (99.1%; median [IQR] age, 27.7 [23.1-32.0] years) delivered after spontaneous conception. In general, infertility treatment increased over time and with maternal age (Table 1). Compared with patients who delivered after spontaneous conception, those who underwent infertility treatment possessed mainly private insurance (239 354 patients [83.2%] vs 15 929 698 patients [51.3%]) and had medium to high income.

**Table 1.** Distribution of Patients With Spontaneous Conception and Conception After Infertility Treatment, Nationwide Readmissions Database (2010-2018)

Characteristic	Patients, No. (%)		
	Total (N = 31 339 991)	Spontaneous conception (n = 31 052 178)	Infertility treatment (n = 287 813)
Delivery year			
2010	3 545 300 (11.3)	3 526 432 (11.4)	18 868 (6.6)
2011	3 503 405 (11.2)	3 481 747 (11.2)	21 657 (7.5)
2012	3 483 983 (11.1)	3 457 496 (11.1)	26 487 (9.2)
2013	3 481 445 (11.1)	3 451 844 (11.1)	29 601 (10.3)
2014	3 496 140 (11.2)	3 460 517 (11.1)	35 624 (12.4)
2015	3 487 848 (11.1)	3 446 451 (11.0)	41 397 (14.4)
2016	3 511 564 (11.2)	3 462 623 (11.2)	48 941 (17.0)
2017	3 440 444 (11.0)	3 407 786 (11.0)	32 658 (11.3)
2018	3 389 862 (10.8)	3 357 282 (10.8)	32 580 (11.3)
Maternal age, y			
<20	2 219 079 (7.1)	2 217 415 (7.1)	1663 (0.6)
20-24	6 885 353 (22.0)	6 867 667 (22.1)	17 687 (6.1)
25-29	9 001 081 (28.7)	8 939 408 (28.8)	61 673 (21.4)
30-34	8 302 425 (26.5)	8 200 125 (26.4)	102 301 (35.5)
35-39	4 002 883 (12.8)	3 931 506 (12.7)	71 376 (24.8)
40-44	871 500 (2.8)	845 131 (2.7)	26 369 (9.2)
≥45	57 669 (0.2)	50 925 (0.2)	6743 (2.3)
Hospital bed size^a^			
Small	4 204 473 (13.4)	4 168 975 (13.4)	35 497 (12.3)
Medium	8 592 312 (27.4)	8 519 156 (27.4)	73 156 (25.4)
Large	18 543 206 (59.2)	18 364 047 (59.1)	179 160 (62.2)
Hospital type			
Government, nonfederal	3 709 228 (11.8)	3 683 472 (11.9)	25 816 (9.0)
Private, not profit	23 705 197 (75.6)	23 456 110 (75.5)	249 087 (86.5)
Private, investment owned	3 925 505 (12.5)	3 912 597 (12.6)	12 909 (4.5)
Teaching hospital status			
Metropolitan, nonteaching	9 634 233 (30.7)	9 573 819 (30.8)	60 415 (21.0)
Metropolitan, teaching	18 497 739 (59.0)	18 282 305 (58.9)	215 433 (74.9)
Nonmetropolitan hospital	3 208 019 (10.2)	3 196 054 (10.3)	11 965 (4.2)
Median household income, quartile^b^			
1 (Low)	8 763 837 (28.0)	8 725 456 (28.1)	38 382 (13.3)
2 (Medium-low)	7 852 931 (25.1)	7 795 731 (25.1)	57 201 (19.9)
3 (Medium-high)	7 752 575 (24.7)	7 676 465 (24.7)	76 110 (26.4)
4 (High)	6 682 596 (21.3)	6 568 498 (21.2)	114 098 (39.6)
Unknown	288 051 (0.9)	286 029 (0.9)	2022 (0.7)
Insurance			
Medicare	230 051 (0.7)	228 184 (0.7)	1867 (0.6)
Medicaid	13 391 040 (42.7)	13 354 568 (43.0)	36 472 (12.7)
Private	16 169 052 (51.6)	15 929 698 (51.3)	239 354 (83.2)
Self-pay	497 498 (1.6)	495 249 (1.6)	2249 (0.8)
Other	986 739 (3.1)	979 090 (3.2)	7649 (2.7)
Unknown	65 611 (0.2)	65 389 (0.2)	221 (0.1)
Comorbidities			
Chronic hypertension	608 744 (1.9)	595 368 (2.0)	13 376 (5.1)
Preeclampsia without severe features	760 204 (2.4)	748 379 (2.5)	11 825 (4.5)
Preeclampsia with severe features	578 521 (2.4)	566 196 (1.8)	12 325 (4.2)
Eclampsia	31 917 (0.1)	31 609 (0.1)	308 (0.1)
Superimposed preeclampsia	237 602 (0.8)	231 610 (0.7)	5993 (2.1)
Fetal growth restriction	921 016 (2.9)	907 029 (2.9)	13 987 (4.7)
Placental abruption	328 805 (1.0)	323 936 (1.0)	4869 (1.7)
Gestational diabetes	981 231 (3.1)	957 141 (2.0)	24 090 (8.2)
Stillbirth	136 161 (0.4)	134 428 (0.4)	1734 (0.6)
Multiple gestation	588 733 (1.9)	546 775 (1.7)	41 958 (14.2)
Cesarean delivery	10 034 901 (31.7)	9 891 702 (31.5)	143 198 (48.5)
Preterm delivery	2 452 649 (7.7)	2 404 940 (7.7)	47 709 (16.2)
Body mass index 30 to <40^c^	723 841 (2.4)	706 535 (2.2)	17 306 (5.9)

^a^ Hospital bed size categories changed by region and hospital type.^35^

^b^ Median household income quartile categories changed each year.^36^

^c^ Body mass index is calculated as weight in kilograms divided by height in meters squared.

The absolute risk of stroke following a pregnancy conceived via infertility treatment was low. The rate of stroke complications requiring hospitalization within 12 months following a delivery was 37 hospitalizations per 100 000 people (105 patients) among patients who underwent infertility treatment and 29 hospitalizations per 100 000 people (9027 patients) among those who did not undergo infertility treatment (rate difference, 8 hospitalizations per 100 000 people; 95% CI, −6 to 21 hospitalizations per 100 000 people) (Table 2). The risk of hospitalization for stroke complications within the calendar year of delivery in relation to infertility treatment status are presented in Table 3. Compared with those who spontaneously conceived, the adjusted HR of any stroke hospitalization within 12 months of delivery was 1.66 (95% CI, 1.17-2.35) for those undergoing infertility treatment (ie, a 66% increased risk of stroke hospitalization). The risk of hemorrhagic stroke hospitalization (adjusted HR, 2.02; 95% CI 1.13-3.61) was larger than that of ischemic stroke (adjusted HR, 1.55; 95% CI, 1.01-2.39) among those who received infertility treatment. In general, when these associations were corrected for biases due to exposure misclassification, selection, and unmeasured confounding, the associations of infertility treatment with risk of hospitalization for hemorrhagic stroke became larger, whereas the corresponding associations for ischemic stroke hospitalizations were attenuated (Table 3).

**Table 2.** Rates of Nonfatal Stroke Hospitalizations Among Patients With Infertility Treatment Procedures and Spontaneous Conceptions, Nationwide Readmissions Database (2010-2018)

Stroke hospitalization type	Patients, No. (rate of hospitalization per 100 000 people)		Rate difference per 100 000 people (95% CI)
	Spontaneous conception^a^	Infertility treatment^b^	
None	30 914 363 (NA)	285 982 (NA)	NA
Any	9027 (29)	105 (37)	8 (−6 to 21)
Hemorrhagic stroke	3791 (12)	52 (18)	6 (−5 to 17)
Ischemic stroke	5734 (19)	55 (19)	1 (−8 to 9)

Abbreviation: NA, not applicable.

^a^ The follow-up period for those who delivered after spontaneous conception was 31 100 181 person-months.

^b^ The follow-up period for those who delivered after infertility treatment was 288 321 person-months.

**Table 3.** Association of Infertility Treatment With Risk of Nonfatal Cardiovascular Complications, Nationwide Readmissions Database (2010-2018)^a^

Cardiovascular event	HR (95% CI)		Bias-corrected rate ratio (95% CI)^c^	E-value HR (95% CI closest to null)
	Unadjusted	Adjusted^b^		
Any stroke	1.76 (1.25-2.48)	1.66 (1.17-2.35)	1.38 (0.86-2.23)	2.71 (1.62)
Hemorrhagic stroke	2.30 (1.31-4.06)	2.02 (1.13-3.61)	3.61 (2.17-5.99)	3.46 (1.51)
Ischemic stroke	1.66 (1.09-2.54)	1.55 (1.01-2.39)	1.39 (0.88-2.26)	2.47 (1.11)

Abbreviation: HR, hazard ratio.

^a^ The median follow-up was 6.3 months (95% CI, 6.3-6.3 months) among those who delivered without infertility treatment and 6.2 months (95% CI, 6.2-6.3 months) among those who delivered with infertility treatment.

^b^ HRs were adjusted for the following confounders: maternal age, age-squared (both centered), multiple births, hospital bed size, hospital type, hospital teaching status, income quartile, insurance, and year of hospital discharge.

^c^ Bias-corrected rate ratios refer to sequential corrections for exposure (infertility treatment) misclassification, selection, and unmeasured confounding (see text for details).

We estimated risk of hospitalizations for stroke (and stroke subtypes) on the basis of discrete time points within the year following delivery (Figure). The adjusted HRs of overall stroke hospitalizations increased as time since the delivery hospitalization increased, and this pattern was particularly evident for hemorrhagic stroke hospitalizations. When these associations were corrected for biases due to exposure misclassification, selection, and unmeasured confounding, the effect size estimates became substantially larger for hemorrhagic stroke hospitalizations and remained similar to the adjusted estimates for ischemic stroke hospitalizations.

**Figure.**
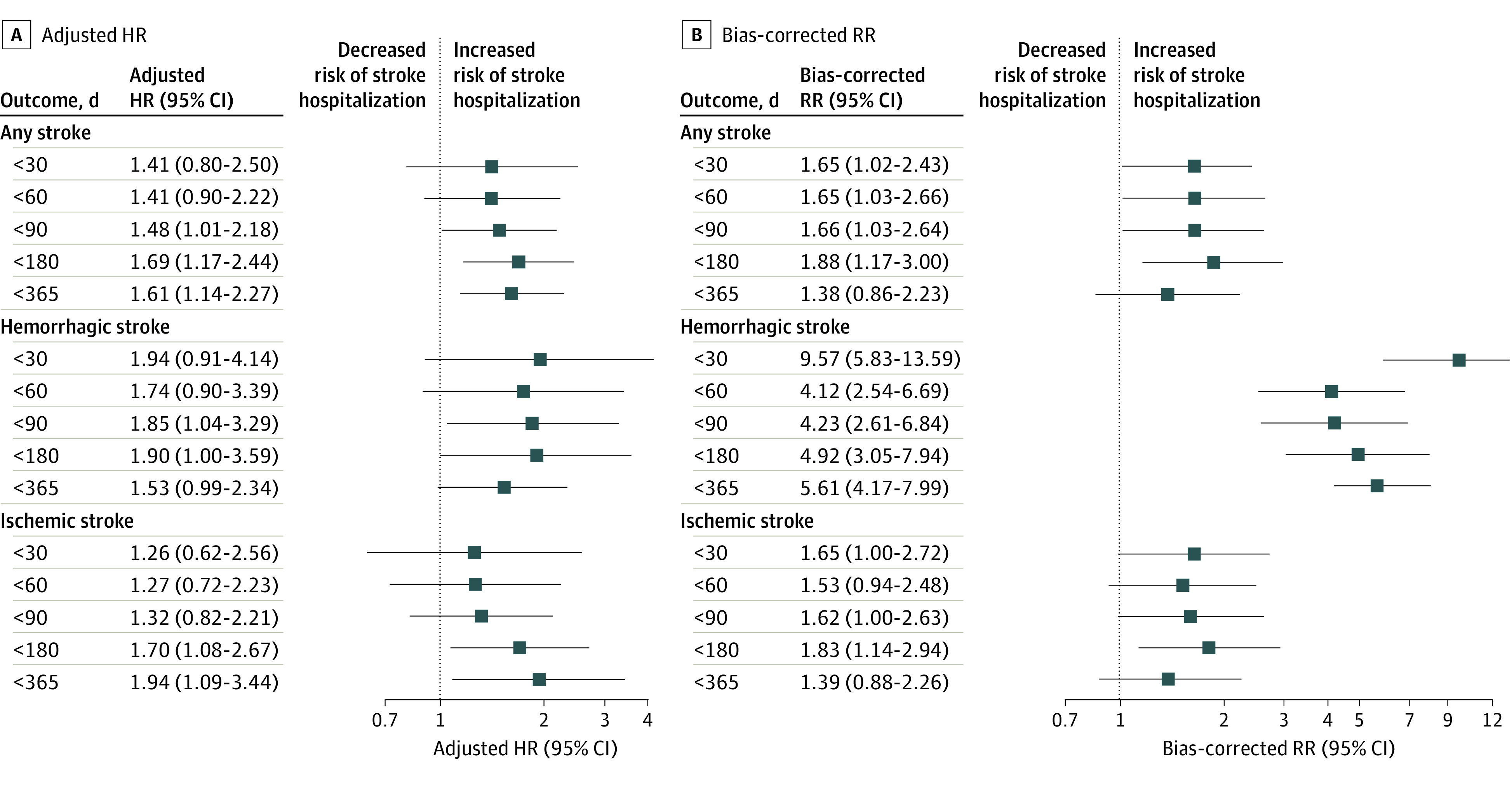
Association of Stroke Hospitalization With Infertility Treatment, Nationwide Readmissions Database (2010-2018) Panel A shows adjusted hazards ratios (HRs), and panel B shows rate ratios (RRs) corrected for exposure misclassification bias, selection bias, and bias due to unmeasured confounders. Squares denote effect size estimates, and error bars denote 95% CIs.

## Discussion

Our large population-based cohort study was warranted, given the lack of definitive conclusions in the current literature regarding the association of infertility treatment with stroke risk. Although the absolute rates of hospitalization were low, we found that infertility treatment was associated with an overall 66% increased risk of stroke hospitalization. This risk was larger for hospitalization for hemorrhagic stroke than ischemic stroke. Additionally, the risk of hospitalization from stroke, either hemorrhagic or ischemic, generally increased with time postdelivery, but the risk was larger for hemorrhagic strokes. Strikingly, the increase in risk was evident even as early as the first 30 days postdelivery, which highlights the need for early and continued follow-up in this population. Corrections for potential biases increased the magnitude of the association of infertility treatment with hospitalization for hemorrhagic stroke.

Early recognition of cardiovascular factors associated with increased risk of stroke among people of reproductive age is paramount. CVD is not only the leading cause of mortality in women globally but is also, alarmingly, the leading cause of maternal mortality in the US.^5,11,37^ Factors associated with increased risk of stroke in the antepartum and postpartum period include hypertensive disorder, preterm delivery, fetal growth restriction, diabetes, and obesity, among many others.^7,11,38^ Of these conditions, hypertensive disorders pose the greatest threat to future stroke risk. A large cross-sectional study^39^ using the 1994 to 2011 Nationwide Inpatient Sample reported a 5.2% increased risk of stroke in patients with a hypertensive disorder during pregnancy. Too et al^40^ also used the NRD to evaluate the risk of readmission for stroke within 60 days after delivery from 2013 to 2014 and found that 14.2% of readmissions for stroke occurred in patients with a history of hypertension; however, most readmissions (81.4%) occurred in patients without this history. These findings suggest that other traditional health factors (ie, obesity, diabetes, and other adverse pregnancy outcomes) and potential unidentified factors are contributing to the risk of stroke. Whether infertility treatment contributes to pregnancy-related stroke hospitalizations has been largely up for debate, with very few large-scale studies examining this association.

Our findings run contrary to a large retrospective cohort study by Ge and colleagues^14^ that concluded infertility medications were associated with a reduced risk of CVD (adjusted HR, 0.83; 95% CI, 0.74-0.94) and ischemic stroke (adjusted HR, 0.82; 95% CI, 0.68-0.99) compared with age-matched controls. The risk of hemorrhagic stroke was not examined.^14^ Udell et al^15^ arrived at a similar conclusion after a large population-based cohort study with median follow-up of 9.7 years. Those who had received infertility treatment had a reduced risk of future CVD (adjusted HR, 0.55; 95% CI, 0.41-0.74), as well as thromboembolic events and all-cause mortality.^15^ Cairncross et al^41^ conducted a longitudinal cohort study of infertility patients and also found no associations of self-reported infertility (with or without treatment) with the development of CVD risk. A meta-analysis^42^ of 5 studies analyzing the association of infertility with stroke had inconclusive findings.

In contrast, Liang et al^43^ conducted a pooled analysis of 8 cohort studies and found an increased risk of nonfatal stroke (HR, 1.14; 95% CI, 1.08-1.20) in patients with infertility. Likewise, a 2019 retrospective cohort study^44^ concluded that infertility was associated with the development of chronic medical conditions, including stroke. These studies suggest that even prior to infertility treatment, this population may already have a baseline increased risk for the future development of CVD and stroke. Infertility treatment itself may then be compounding this risk further. Baldini et al^45^ argue that given the increasing use of ART with age, as well as the increased risk of adverse pregnancy outcomes with age, older individuals should have a thorough CVD risk assessment even prior to receiving ART. Perhaps this recommendation should be extended further to all individuals prior to receiving any type of infertility treatment.

We posit 3 pathways to explain the association of infertility treatment with stroke. First, infertility treatment may contribute to certain vascular complications, such as ischemic placental disease (eg, preeclampsia, placental abruption, and fetal growth restriction), as well as kidney disorders and metabolic aberrations (eg, preexisting and gestational diabetes).^46,47^ These complications, in turn, may increase stroke risk.^15,18,48,49,50,51^ In a longitudinal cohort study over 18 years, Fraser et al^18^ found that hypertension in pregnancy and gestational diabetes, were associated with a 10-year increased risk of CVD based on the Framingham prediction score.

Second, it is possible that certain physiologic changes, endothelial damage, or induction of a prothrombotic state and alterations in maternal hemodynamics at the time of infertility treatment are contributing to the increase in risk of stroke.^15,19,20,52^ Fujitake et al^52^ found changes in heart rate and blood pressure at various time points during stimulation, especially when the agonist protocol was used compared with the antagonist protocol. However, it remains uncertain whether these changes are associated with future adverse pregnancy outcomes or pregnancy-related stroke hospitalization.

Third, people receiving infertility treatment may already have certain health factors known to be associated with an increased risk in stroke (eg, having overweight or obesity, smoking, or alcohol use), and pregnancy itself may unmask these shared risk factors. Although it is beyond the scope of this article to determine which of these pathways would best explain the association of infertility treatment with stroke rehospitalizations, the examination of these issues is worthy of future investigations.

### Strengths and Limitations

A major strength of our study was the use of a large readmissions database that encompasses many states and hospitalizations over a prolonged period. This allows our results to be generalizable to a broad population. By computing a sensitivity analysis, we provided reassurance of the unlikelihood of unmeasured confounders obscuring our adjusted HRs and ensured the robustness of our associations.

This study also has limitations. By restricting the data to *ICD-9*-*CM* or *ICD-10-CM* codes, we could not differentiate between types of infertility treatment (ie, IVF vs intrauterine insemination). Additionally, pregnancy resulting from infertility treatment may be underreported in the database because it relies on proper coding. We could not control specifically for several factors that may contribute to or modify the association of infertility treatment with stroke, including prepregnancy hypertension or diabetes, hypertensive disorder of pregnancy, gestational diabetes, fetal growth restriction, stillbirth, or preterm birth. Likewise, there is a lack of information on management of certain medical conditions, including hypertension and diabetes. Additionally, we were unable to examine these associations by maternal race or ethnicity owing to a lack of data. Small increases in the risk of stroke hospitalization for different types of infertility treatment may have resulted in exceedingly large HRs (particularly for the bias-corrected associations for hemorrhagic stroke). Therefore, the effect size estimates should be cautiously interpreted.

Although a few potential mechanisms associating infertility treatment with the increased risk of stroke were previously described, there are additional unknown factors that could not be accounted for in this study. For example, subarachnoid hemorrhage is considered a type of hemorrhagic stroke; undetected aneurysms or arteriovenous malformations could increase in size during infertility treatment and pregnancy, suggesting that women at risk of aneurysm or arteriovenous malformation may need more targeted testing prior to infertility treatment. Further exploration of such factors is needed in future studies.

## Conclusions

Because the previous literature had conflicting conclusions regarding the association of infertility treatment with stroke risk, we performed this large population-based study using the NRD. Our findings of an increased stroke risk in patients undergoing infertility treatment up to 12 months after delivery deserve immediate attention given that maternal mortality is associated with CVD. Further exploration into this association is warranted and efforts should be made to mitigate all factors associated with increased risk of stroke and other CVD in the antenatal and postpartum periods. The American College of Obstetricians and Gynecologists currently recommends routine follow-up within 3 weeks post partum, with an additional follow-up within 12 weeks post partum.^53^ Earlier follow-up is recommended for certain patients at high risk for postpartum complications, such as those with hypertensive disorders of pregnancy, where follow-up is recommended between 7 and 10 days post partum.^11^ Given the association of infertility treatment with increased risk of hemorrhagic and ischemic strokes, perhaps we should consider early and continued follow-up for patients who undergo infertility treatment.

## Notes

Supplement 1. **eTable.** *International Classification of Disease* Codes Used in the Study

Supplement 2.
Data Sharing Statement
