# Supplementary material for: Risk of Stroke Hospitalization After Infertility Treatment
Source: JAMA Netw Open. 2023 Aug 30;6(8):e2331470. doi: 10.1001/jamanetworkopen.2023.31470 (PMC10469284; doi:10.1001/jamanetworkopen.2023.31470)
Supplement: Supplement 1. — eTable. International Classification of Disease Codes Used in the Study [file jamanetwopen-e2331470-s001.pdf]

Supplemental Online Content

Sachdev D, Yamada R, Lee R, Sauer MV, Ananth CV. Risk of stroke hospitalization after infertility treatment. *JAMA Netw Open*. 2023;6(8):e2331470. doi:10.1001/jamanetworkopen.2023.31470

eTable. *International Classification of Disease* Codes Used in the Study

This supplemental material has been provided by the authors to give readers additional information about their work.

© 2023 Sachdev D et al. *JAMA Network Open.*

eTable
***International Classification of Disease* Codes Used in the Study**

Description	ICD-9	ICD-10
**Inclusion Criteria**		
Delivery	V27, 650, 720*, 721*, 7221*, 7229*, 7231*, 7239*, 724*, 726*, 7251*, 7252*, 7253*, 7254*, 7271*, 7279*, 728*, 729*, 7322*	Z37, O80, 10D07Z3*, 0W8NXZZ*, 10D07Z4*, 10D07Z5*, 10S07ZZ*, 10D07Z3*, 10D07Z4*, 10D07Z5*, 10D07Z6*, 0W8NXZZ*, 10D07Z6*, 10D07Z8*, 10D07Z7*,
Caesarean Section	649.8, 669.70, 669.71, 740*, 741*, 742*, 744*, 7499*	O82, O75.82 10D00Z0*, 10D00Z1*, 10D00Z2*
Multiple Births	V27.2-V27.7, 651	Z37.2-Z37.7, O30
**Exclusion Criteria**		
Ectopic and Molar Pregnancy and other pregnancies with abortive outcomes	630-639 6901*, 6951*, 7491*, 750*	O00, O01, O02, O03, O04, O07, O08, Z33.2 10A07ZZ*, 10A08ZZ*, 10A00ZZ*, 10A03ZZ*, 10A04ZZ*, 10A07ZX*
**Assisted reproductive technology (ART) Diagnosis Codes**		
**Encounter for other procreative management**		
Encounter for male factor infertility in female patient	Z31.8	V26.89
**Encounter for gamete intrafallopian transfer (GIFT)**	Z31.81	V26.81
**Encounter for assisted reproductive fertility procedure cycle**	Z31.83 Z31.83	V26.81
Encounter for fertility preservation procedure	Z31.84	V26.82
**Encounter for other procreative management (IUI cycle, artificial insemination)**	Z31.89	V26.1/V26.89
Encounter for procreative management and counseling for gestational carrier	Z31.7	V26.89
Encounter for procreative management, unspecified	Z31.9	V26.9
**Procreation management investigation and testing**		
Fertility testing	Z31.4	V26.2
Aftercare following sterilization reversal	Z31.41	V26.21
Encounter for other procreative investigation/testing	Z31.42	V26.22
Encounter for general counseling and procreation advice	Z31.49	V26.29
Procreative counseling/advice using natural family planning	Z31.6	
Encounter for fertility preservation counseling	Z31.61	V26.41
Encounter for other general counseling and advice on procreation	Z31.62 Z31.69	V26.42 V26.49

© 2023 Sachdev D et al. *JAMA Network Open.*

Description	ICD-9	ICD-10
**Pregnancy resulting from ART**	O09.81	V23.85
Unspecified trimester	O09.819	V23.85
In first trimester	O09.811	V23.85
In second trimester	O09.812	V23.85
In third trimester	O09.813	V23.85
**Supervision of pregnancy with history of infertility**	O09.0	V23.0
Unspecified trimester	O09.00	V23.0
First trimester	O09.01	V23.0
Second trimester	O09.02	V23.0
Third trimester	O09.03	V23.0
**Complications associated with artificial fertilization**	N98	-
Infection associated with artificial insemination	N98.0	-
Hyperstimulation of ovaries	N98.1	256.1
Complications of attempted IVF	N98.2	-
Complications of embryo transfer	N98.3	-
Other complications associated with artificial fertilization	N98.8	-
Disorder due to artificial insemination	N98.9	-
Exam of potential donor of organ or tissue	Z00.5	V70.8
Egg Donor		
Egg Donor, unspecified	Z52.819	V59.70
Egg Donor, < 35, anonymous	Z52.810	V59.71
Egg Donor, <35, designated	Z52.811	V59.72
Egg Donor, 35+, anonymous	Z52.812	V59.73
Egg Donor, 35+, designated	Z52.813	V59.74
Female Infertility associated with anovulation	N97.0	628.0
Female Infertility of hypothalamic pituitary origin	E23.0	628.1
Female Infertility of tubal origin	N97.1	628.2
Female Infertility of uterine origin	N97.2	628.3
Female Infertility of cervical/vaginal origin	N97.8	628.4
Female Infertility of other origin	N97.8	628.8
Female Infertility, unspecified	N97.9	628.9
Female Infertility associated with Stein-Leventhal Syndrome	E28.2	256.4
Tuboplasty, post previous sterilization	Z31.0	V26.0
**Stroke (any)**	430-438	I60-I69
Ischemic stroke	433-437	I63, I65-I67
Hemorrhagic stroke	430-432	I60-I62, I69

*Procedure Codes. Delivery-related procedures for forceps, breech extraction, vacuum extraction, other specified and unspecified delivery, and internal and combined version and extraction. Exclusion criteria procedures for pregnancy with abortive outcomes.

© 2023 Sachdev D et al. *JAMA Network Open.*
