# Supplementary material for: Risk of Stroke Hospitalization After Infertility Treatment
Source: JAMA Netw Open. 2023 Aug 30;6(8):e2331470. doi: 10.1001/jamanetworkopen.2023.31470 (PMC10469284; doi:10.1001/jamanetworkopen.2023.31470)
Supplement: Supplement 2. — Data Sharing Statement [file jamanetwopen-e2331470-s002.pdf]

# Data Sharing Statement

Sachdev. Risk of Stroke Hospitalization After Infertility Treatment. *JAMA Netw Open*.
Published August 30, 2023. doi:10.1001/jamanetworkopen.2023.31470

**Data**
**Data available:** No

**Additional Information**
**Explanation for why data not available:** The data used in this study were obtained from the Agency for Health Care and Quality's Healthcare Cost and Utilization Project (HCUP), available at https://www.hcup-us.ahrq.gov/nrdoverview.jsp
